# Apalutamide and Stereotactic Body Radiotherapy in Metastatic Hormone-Sensitive Prostate Cancer: Multicenter Real-World Study

**DOI:** 10.3390/cancers17132216

**Published:** 2025-07-02

**Authors:** Juan A. Encarnación, Virginia Morillo Macías, Isabel De la Fuente Muñoz, Violeta Derrac Soria, Luis Fernández Fornos, María Albert Antequera, Osamah Amr Rey, Vicente García Martínez, José L. Alonso-Romero, Raquel García Gómez

**Affiliations:** 1 Department Radiation Oncology, Hospital Clínico Universitario Virgen de la Arrixaca, 30120 Murcia, Spain; isabeldelafuente123@gmail.com; 2 Faculty of Medicine, University of Murcia, 30100 Murcia, Spain; 3 Murcian Institute of Biosanitary Research, 30120 Murcia, Spain; 4 Department Radiation Oncology, Consorcio Hospitalario Provincial, 12002 Castellón, Spain; vmorill@gmail.com; 5 Department Radiation Oncology, Hospital Universitario La Ribera, 46600 Alzira, Spain; 6 Department Radiation Oncology, Hospital Universitario San Juan, 03550 Alicante, Spain; lferfor@gmail.com; 7 Department Radiation Oncology, Consorcio Hospital General Universitario, 46014 Valencia, Spain; maria2albert@gmail.com; 8 Department Radiation Oncology, Hospital Clínico Universitario, 46010 Valencia, Spain; oamrey@gmail.com; 9 Department Radiation Oncology, Hospital General Universitario Santa Lucía, 30202 Cartagena, Spain; vgm20@hotmail.com; 10 Department of Medical Oncology, Hospital Clínico Universitario Virgen de la Arrixaca, 30120 Murcia, Spain; josel.alonso2@carm.es; 11 Department Radiation Oncology, Hospital Universitario y Politécnico La Fe, 46026 Valencia, Spain

**Keywords:** metastatic prostate cancer, apalutamide, stereotactic body radiotherapy, SBRT, androgen receptor signaling inhibitors, real-world study, PSA response, metastasis-directed therapy, oligometastatic disease

## Abstract

Background: The management of metastatic hormone-sensitive prostate cancer (mHSPC) has evolved with the integration of androgen receptor signaling inhibitors (ARSIs) and metastasis-directed therapies (MDTs). Stereotactic body radiotherapy (SBRT) offers precise local control, yet real-world data on its combination with apalutamide remain limited. Methods: We conducted a multicenter retrospective cohort study including 134 patients with mHSPC treated with apalutamide and SBRT between February 2021 and December 2024. The primary endpoints were progression-free survival (PFS), local control (LC), and treatment safety. PSA kinetics and radiologic response were evaluated, and outcomes were analyzed according to PSA thresholds and treatment timing. Results: Most patients (93.3%) had low-volume disease; 97.1% presented with ≤5 metastases. At a median follow-up of 28 months, LC was 99.3% and 95.5% of patients were progression-free. Complete radiological response was achieved in 87.5% of patients, and 68.4% attained ultralow PSA levels (≤0.02 ng/mL). Undetectable PSA and radiologic complete response were independently associated with improved PFS (*p* = 0.010 and *p* = 0.011, respectively). Treatment was well tolerated, with grade ≥3 toxicity occurring in only 2.2% of patients. Conclusions: The combination of apalutamide and SBRT in mHSPC is associated with high local and systemic disease control and minimal toxicity in a real-world setting. This approach may delay systemic treatment intensification and the onset of castration resistance. Prospective studies are warranted to confirm these findings.

## Simple Summary

Metastatic prostate cancer is a serious condition with limited treatment options that offer long-term control. In recent years, new oral hormonal therapies have improved outcomes in patients with hormone-sensitive metastatic disease. Additionally, focused radiation techniques such as stereotactic body radiotherapy (SBRT) have been used to target individual cancer lesions. However, it is still unclear how these strategies work together in real-life clinical practice. This study evaluated the combination of a hormonal therapy called apalutamide with SBRT in patients from multiple centers across Spain. We observed high rates of tumor control and very low levels of side effects. Our results suggest that combining these treatments may delay the need for more aggressive therapies and help patients maintain a better quality of life. These findings support the use of this approach in selected patients with metastatic prostate cancer.

## 1. Introduction

Prostate cancer (PC) is one of the most common malignancies among men worldwide, representing a major public health burden due to its high incidence and the increasing life expectancy of the population [1,2]. In Europe, it accounts for approximately one in ten new cancer cases, and in Spain, although the incidence exceeds 12%, the majority of patients exhibit a high five-year survival rate, reflecting the efficacy of treatments in localized stages [3,4]. Approximately 4% of cases are diagnosed at the metastatic stage, either synchronously or metachronously, which is associated with a poorer prognosis [5,6].

In metastatic hormone-sensitive prostate cancer (mHSPC), the response to treatment is heterogeneous and depends on both clinical and biological factors [7]. Stratification based on tumor burden and the timing of metastasis onset has shown prognostic relevance [8,9]. These distinctions have contributed to the development of individualized therapeutic strategies aimed at optimizing clinical outcomes [10]. Historically, androgen deprivation therapy (ADT) monotherapy has been the standard treatment for these patients. However, progression to castration-resistant disease in a proportion of cases has driven the adoption of combination therapeutic approaches [11,12].

Over the past decade, the treatment landscape has evolved significantly with the introduction of androgen receptor signaling inhibitors (ARSIs), which have demonstrated improvements in overall survival (OS) and progression-free survival (PFS) when administered in combination [13,14,15,16,17,18,19,20].

Despite the advances observed in clinical trials, their implementation in real-world practice continues to present challenges. Factors such as comorbidities, advanced age, patient preferences, and regional disparities in access to therapies may influence both treatment selection and effectiveness. Moreover, the limited representation of certain subgroups in pivotal trials restricts the generalizability of the findings [15,16,17,18,19].

In this context, it is essential to complement clinical trial data with observational studies that reflect the effectiveness of treatments in routine clinical practice. This type of evidence is key to optimizing therapeutic sequencing, improving patient selection, and reducing the gap between clinical guidelines and real-world care. Specifically, in the management of mHSPC, stereotactic body radiation therapy (SBRT) has emerged as a relevant therapeutic option for patients with oligometastatic disease, allowing effective local control (LC) of metastases with an acceptable toxicity profile [21,22,23].

The objective of the present study is to evaluate the efficacy and safety of metastasis-directed therapy (MDT) using SBRT in mHSPC patients treated with apalutamide. Through a retrospective cohort analysis, we aim to assess the impact of SBRT as a complementary strategy in the management of metastatic disease, with the goal of delaying the initiation of a new systemic therapy (SST) line. This study aims to address this gap by evaluating the real-world impact of SBRT combined with apalutamide in a population with heterogeneous metastatic burden, thereby exploring an intermediate therapeutic approach between systemic intensification alone and MDT in isolation.

## 2. Materials and Methods

### 2.1. Study Design

Following approval by the ethics committee, a retrospective observational study was conducted, including mHSPC patients treated with apalutamide who also received MDT. Apalutamide was selected as the androgen receptor signaling inhibitor for this study because it is the most widely used agent in our clinical setting for patients with metastatic hormone-sensitive prostate cancer. Consequently, the cohort reflects real-world clinical practice in our region. All eligible patients from the participating centers who received both apalutamide and metastasis-directed SBRT for metastatic hormone-sensitive prostate cancer. The inclusion period was from February 2021 to December 2024, and data were collected at 8 centers nationwide.

### 2.2. Data Collection

Clinical data were extracted from electronic medical records, and follow-up was maintained until the end of the observation period or death, whichever occurred first. Collected variables were categorized into baseline characteristics and clinical outcomes following MDT.

Baseline data included age, prostate-specific antigen (PSA) level, type and location of metastases, date of apalutamide initiation, and time from apalutamide initiation to SBRT administration.

Treatment outcomes included LC, PFS, OS, toxicity (classified according to CTCAE v5.0), and PSA kinetics following treatment.

PSA values were collected retrospectively from electronic medical records. There was no standardized prospective protocol in place to define specific time points for PSA measurement. However, in the majority of cases, PSA levels were available at key clinical milestones: at the initiation of apalutamide, approximately one month after treatment initiation, prior to metastasis-directed radiotherapy, and during follow-up visits—typically every three months. These time points were selected for analysis because they represented the most consistently documented measurements across participating centers. Comparisons were made between groups based on disease volume, metastasis location, and number of lesions treated. Further stratification was conducted according to the time interval between apalutamide initiation and SBRT (≤ or >3 months). LC was defined as the absence of radiological progression in treated lesions, assessed by conventional imaging or next-generation imaging (NGI), based on the initial diagnostic imaging modality (PET/CT, CT, bone scan). In patients with de novo oligometastatic disease, the primary prostate tumor was treated with radiotherapy in all cases. This uniform approach was intended to optimize local control and is consistent with current evidence supporting the treatment of the primary tumor in the oligometastatic setting.

Toxicity data were collected retrospectively through a systematic review of electronic medical records at all participating centers. The evaluation was based on physician-reported adverse events documented during follow-up visits, and grading was assigned retrospectively according to CTCAE v5.0 criteria. Toxicity reporting was performed uniformly across centers following a standardized data collection template designed for the study.

### 2.3. Statistical Analysis

A descriptive analysis of baseline characteristics, progression patterns, and clinical events post-treatment was performed. Categorical variables were expressed as absolute frequencies and percentages, while continuous variables were summarized using medians and interquartile ranges.

PFS, LC, and OS were estimated using the Kaplan–Meier method and compared using the log-rank test. Cox regression models were employed to explore associations between clinical factors (metastatic burden, location, time to progression, PSA levels) and outcomes of interest. A *p*-value of <0.05 was considered statistically significant. Data analysis was conducted using SPSS for Windows, version 25.0 (IBM Corp. Armonk, NY, USA).

## 3. Results

### 3.1. Baseline Characteristics

The study cohort included 134 patients with mHSPC, the majority of whom were diagnosed using next-generation imaging (NGI) techniques (83.8%). Most patients presented with low-volume metastatic disease (93.3%). A detailed description of baseline characteristics is provided in Table 1.

### 3.2. Lesion Distribution and Characteristics of Metastatic Disease

A total of 97.1% of patients presented with fewer than five lesions at diagnosis, although not all lesions were necessarily treated. Not all metastatic lesions were treated because some patients presented with multiple metastases, and the approach to SBRT varied across participating centers. In certain institutions, the clinical decision was made to target only selected lesions based on factors such as lesion size, location, symptomatology, and institutional protocols. Bone lesions accounted for 62.7% of SBRT treatments, extrapelvic nodal metastases for 29.1%, and visceral metastases for 3%. A single lesion was treated in 66.2% of patients, two lesions in 22.8%, three lesions in 8.8%, and four lesions in 2.2%.

### 3.3. Fractionation Scheme

The distribution of dose per fraction was analyzed, with the most common regimen being 10 Gy in 3 fractions (30.1%), followed by 9 Gy in 3 fractions (16.9%) and 7.5 Gy in 6 fractions (12.5%). All fractionation schemes used delivered a Biologically Effective Dose (BED) greater than 100 Gy.

### 3.4. PSA Levels

A PSA reduction of more than 90% from baseline during the first follow-up after ARSI initiation was achieved in 21.3% of patients. The median PSA level before SBRT was 0.96 ng/mL (range: 0.01–140 ng/mL), and a median PSA of 0.06 ng/mL was observed following metastasis-directed therapy.

In 12.5% of patients, no PSA response was observed after ARSI initiation; however, among these, 47% achieved a >90% PSA response following the addition of MDT.

Patients were stratified based on whether they achieved ultralow PSA levels, defined as UL1 (0.02–0.2 ng/mL) and UL2 (≤0.02 ng/mL), in an effort to closely monitor those who did not reach UL2 values. At 28 months of follow-up, 68.4% of patients had reached UL2 levels and 25.7% UL1.

### 3.5. PFS, LC, and OS

The median follow-up was 28 months. Following combination treatment, 87.5% of patients achieved complete response (CR), 10.3% partial response (PR), and 2.2% stable disease (SD). A radiological response was observed in 99.3% of patients at the early assessment (3 months) after MDT. Radiological response was assessed using the same imaging modality initially employed to detect the metastatic lesions in the majority of patients, most commonly PET-CT or bone scintigraphy. Response was evaluated based on radiological criteria, defined as complete disappearance of the treated lesion(s) for complete response, a reduction in lesion size or metabolic activity for partial response, and no significant change for stable disease. 

In PFS analysis, 95.5% of patients remained progression-free at the time of data cutoff. LC was 99.3%, with only one patient showing persistent disease after MDT.

Treatment response was identified as an independent prognostic factor for disease progression, with a 13-fold increased risk in patients who did not achieve CR (HR 13.144, 95% CI: 1.804–95.781; *p* = 0.011).

PFS was significantly longer in patients who achieved undetectable PSA levels (≤0.02 ng/mL), reaching 44.68 months (95% CI: 44.02–45.29), compared to 42.51 months (95% CI: 39.57–46.20) in those with PSA > 0.02 ng/mL (*p* = 0.010).

Achieving a UL2 PSA level was associated with improved OS (HR 3.095, 95% CI: 0.868–11.039; *p* = 0.082), showing a trend without reaching statistical significance. In multivariate analysis, UL2 PSA levels were identified as an independent prognostic factor for disease progression (HR 9.949, 95% CI: 1.158–85.469; *p* = 0.036).

**Figure 1:**
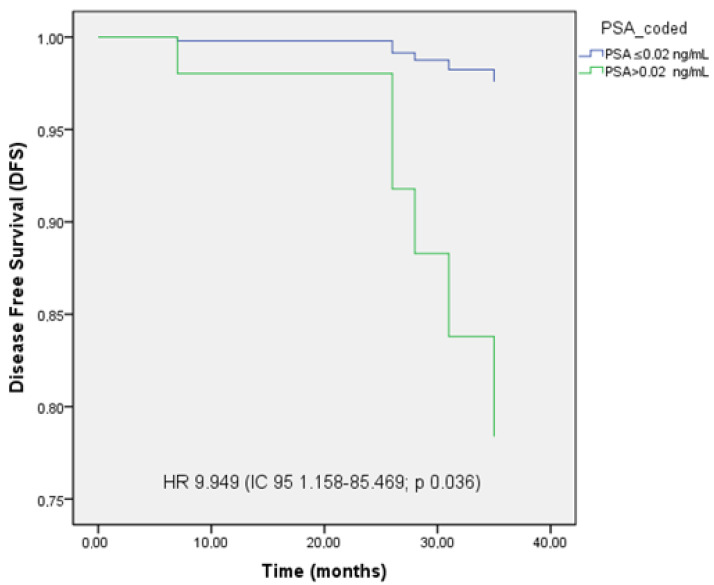
Kaplan–Meier curve of disease-free survival (DFS) stratified by PSA levels ≤ 0.02 ng/mL and >0.02 ng/mL.

**Figure 2:**
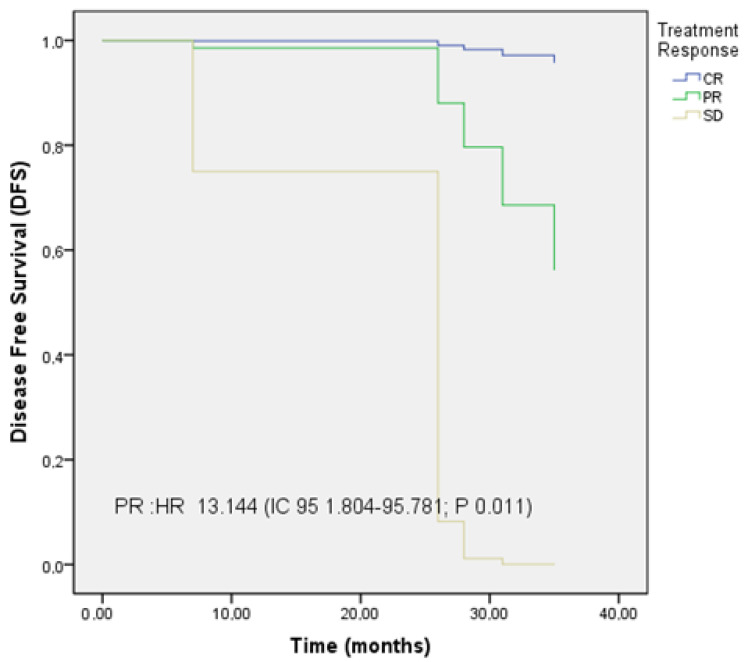
Kaplan–Meier curves of disease-free survival (DFS) according to treatment response.

**Figure 3:**
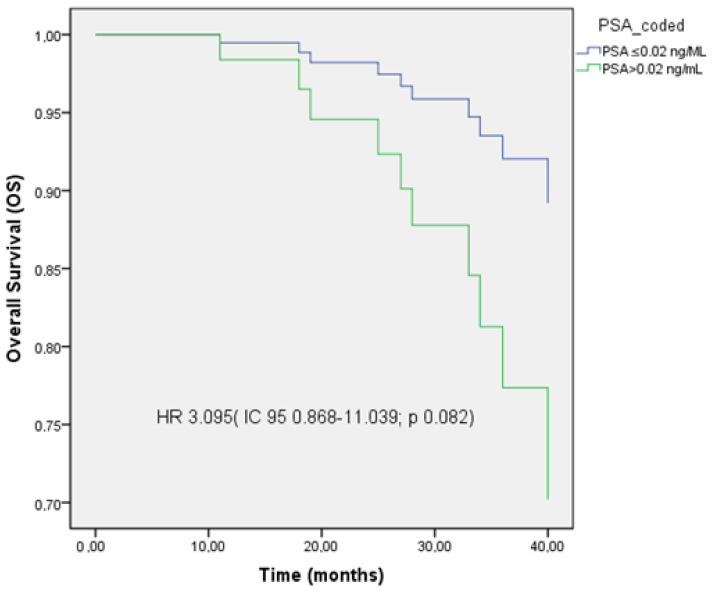
Kaplan–Meier curve of overall survival (OS) stratified by PSA levels ≤ 0.02 ng/mL and >0.02 ng/mL.

The Kaplan–Meier curves illustrating disease-free survival (DFS) and OS are shown in Figure 1, Figure 2 and Figure 3.

### 3.6. Acute and Chronic Toxicity After MDT

Only 2.2% of patients experienced grade 3 (G3) acute toxicity, while G1 and G2 toxicity were observed in 22.1% and 8.1% of cases, respectively. Chronic toxicity was reported as G1 in 14.2% and G2 in 2.2%, with G3 events (2.2%) predominantly consisting of asthenia (1.5%) and pain (0.7%).

## 4. Discussion

In this multicenter retrospective study, we aimed to evaluate the synergistic effect of SBRT combined with ARSI in patients with mHSPC, without the emergence of significant toxicity when both therapies are administered together.

In a clinical context where optimizing treatment sequencing is crucial to prolong systemic therapy efficacy, our findings suggest that metastasis-directed therapy (MDT) via SBRT may provide meaningful clinical benefits. These include delaying the initiation of further systemic therapies and potentially postponing the onset of castration resistance. This approach aligns with the findings of the SABR-COMET trial [24], which demonstrated that SBRT in oligometastatic patients not only improves local control but is also associated with extended overall survival, supporting its role as an effective component of comprehensive metastatic disease management.

A key finding in our cohort was the high disease control rate. At three months post-treatment, 87.5% of patients achieved CR, a proportion that remained stable throughout follow-up. This suggests a substantial systemic and local impact of the combined therapeutic strategy. Moreover, the marked PSA decline following SBRT highlights the potential of MDT to intensify treatment in high-risk patients by reducing tumor clone burden.

Subgroup analysis identified both early clinical response and post-treatment PSA levels as independent prognostic factors. Patients achieving CR demonstrated significantly longer disease-free survival (DFS) compared to those with partial response or stable disease, emphasizing the clinical utility of early response as an efficacy marker. Additionally, PSA levels after SBRT were significantly correlated with outcomes; a PSA ≤ 0.02 ng/mL was associated with improved DFS and OS. These results support PSA not only as a marker of therapeutic response but also as a prognostic indicator following MDT, consistent with prior studies identifying PSA < 0.02 ng/mL as predictive of disease evolution [25,26,27].

Our study demonstrated excellent local control, reinforcing SBRT as a precise and effective modality for targeting progressive metastatic lesions. In the EXTEND study [28], combining systemic therapy with MDT in oligometastatic mHSPC improved radiographic PFS (rPFS) compared to systemic therapy alone, with benefits in PFS and delayed systemic treatment intensification—without a significant increase in toxicity. Although our event numbers are limited, our results are encouraging and support the use of SBRT as a strategy to prolong the duration of systemic response.

It is also noteworthy that most of the patients in our cohort had low-volume disease and predominantly bone metastases, which may have contributed positively to both local control and reduced toxicity. These findings are consistent with the literature, which identifies patients with low-volume disease as those most likely to benefit from local intensification strategies without altering systemic therapy [29,30,31], as exemplified by the PERSIAN trial [32] evaluating SBRT in oligometastatic HSPC.

In terms of safety, the observed toxicity profile was favorable, with most adverse events being mild to moderate, indicating good tolerability of the treatments in this patient population. This toxicity profile is similar to that reported in SBRT-treated patients with castration-resistant prostate cancer (CRPC) [33].

The combination of SBRT and hormonal therapy represents an emerging therapeutic strategy in mHSPC. The synergy lies in SBRT’s ability to eradicate visible metastases while hormonal therapy targets undetectable micrometastatic disease, optimizing overall disease control. The RADIOSA trial [34] was the first randomized study in oligometastatic mHSPC to demonstrate a significant improvement in PFS with the addition of short-term ADT to SBRT compared to SBRT alone. However, it also suggests that carefully selected patients may benefit from SBRT monotherapy, highlighting the importance of individualized treatment selection. This approach could allow for initial SBRT alone, postponing or reducing the duration of systemic therapy and avoiding its cumulative toxicity.

Moreover, the potential immunomodulatory effect of SBRT, through mechanisms such as the abscopal effect and increased antigen presentation, could complement systemic treatments by enhancing anti-tumor responses beyond the irradiated sites. These biological interactions may partially explain the sustained disease control observed in a substantial proportion of our cohort. The favorable safety profile of SBRT further reinforces its role as a viable option, particularly in patients with limited disease burden and long disease-free intervals. Taken together, these findings support the rationale for integrating SBRT into the therapeutic algorithm of mHSPC, not only as a palliative measure but as a disease-modifying strategy within a multimodal approach.

The Wolverine meta-analysis [35], which pooled data from multiple randomized trials, supports the benefit of MDT across several survival-related outcomes in oligometastatic mHSPC. This combined analysis showed improved PFS with MDT compared to no MDT, regardless of whether patients presented de novo, the staging modalities used, castration sensitivity status, or the inclusion of ADT.

The main limitations of our study include its retrospective design and lack of a control group, limiting the ability to establish direct causality. Ongoing studies, such as the phase II Trial of Stereotactic Body Radiation Therapy and Androgen Deprivation for Oligometastases in Prostate Cancer (SBRT-SG 05) [36], aim to address these questions. Additionally, the limited number of progression events in our cohort restricts the statistical power for more robust multivariate analyses. Nevertheless, the multicenter design and inclusion of a cohort representative of real-world clinical practice lend strength and practical applicability to our findings.

Although our findings suggest favorable oncologic outcomes, the retrospective nature of the study and absence of a control group limit the ability to draw causal conclusions, particularly regarding survival benefit. Attempts to construct a matched or historical control cohort were not feasible due to variability in treatment approaches and incomplete data in non-SBRT populations. Therefore, our results should be interpreted as hypothesis-generating and reflective of real-world practice.

Nevertheless, the multicenter design and inclusion of a cohort representative of real-world clinical practice lend strength and practical applicability to our findings. The consistency of clinical benefit observed across participating centers reinforces the external validity of the results and supports the feasibility of incorporating metastasis-directed therapy into routine management of mHSPC.

## 5. Conclusions

SBRT appears to be an effective and well-tolerated therapeutic option in patients with mHSPC treated with apalutamide. Our results reinforce the synergy between both treatments, showing limited toxicity while potentially delaying the onset of castration resistance and the initiation of new systemic therapies. This may positively impact both disease control and patient quality of life. These findings support the value of MDT as a complementary tool in the personalized management of mHSPC and underscore the need for prospective studies to validate these outcomes in larger cohorts.

## Figures and Tables

**Table 1:** Baseline characteristics of patients.

	*n*: 134
Median age at the start of treatment	73 years (56–87)
Type of patient (proportion, *n*)	
Synchronous debut	24.6% (33)
Metachronous biochemical recurrence	75.4% (101)
Diagnostic PSA (median)	8.38 ng/mL (0.25–158)
Metastasis location (proportion, *n*)	
Extrapelvic nodal (M1a)	26.8% (36)
Bone (M1b)	68.7% (92)
Visceral (M1c)	4.5% (6)

## Data Availability

The data presented in this study are available on reasonable request from the corresponding author. The data are not publicly available due to privacy and ethical restrictions.

## Abbreviations

ADT	Androgen Deprivation Therapy
ARSI	Androgen Receptor Signaling Inhibitors
BED	Biologically Effective Dose
BS	Bone Scan
CR	Complete Response
CT	Computed Tomography
CTCAE	Common Terminology Criteria for Adverse Events
Fig	Figure
G	Grade
LC	Local Control
mHSPC	Metastatic Hormone-Sensitive Prostate Cancer
MDT	Metastasis-Directed Therapy
OS	Overall Survival
PET/CT	Positron Emission Tomography/Computed Tomography
PC	Prostate Cancer
PR	Partial Response
PSA	Prostate Specific Antigen
PFS	Progression-Free Survival
rPFS	Radiographic Progression–Free Survival
SBRT	Stereotactic Body Radiation Therapy
SD	Stable Disease
SST	Subsequent Systemic Therapy
UL1 PSA	Ultralow 1 Prostate Specific Antigen
UL2 PSA	Ultralow 2 Prostate Specific Antigen
